# Influence of hypothermia on right atrial cardiomyocyte apoptosis in patients undergoing aortic valve replacement

**DOI:** 10.1186/1749-8090-2-7

**Published:** 2007-01-23

**Authors:** Evaristo Castedo, Raquel Castejón, Emilio Monguio, Sebastian Ramis, Carlos G Montero, Santiago Serrano-Fiz, Raul Burgos, Cristina Escudero, Juan Ugarte

**Affiliations:** 1 Department of Cardiothoracic Surgery, Clinica Puerta de Hierro, Madrid, Spain; 2 Laboratory of Internal Medicine, Clinica Puerta de Hierro, Madrid, Spain

## Abstract

**Background:**

There is increasing evidence that programmed cell death can be triggered during cardiopulmonary bypass (CPB) and may be involved in postoperative complications. The purpose of this study was to investigate whether apoptosis occurs during aortic valve surgery and whether modifying temperature during CPB has any influence on cardiomyocyte apoptotic death rate.

**Methods:**

20 patients undergoing elective aortic valve replacement for aortic stenosis were randomly assigned to either moderate hypothermic (ModHT group, n = 10, 28°C) or mild hypothermic (MiHT group, n = 10, 34°C) CPB. Myocardial samples were obtained from the right atrium before and after weaning from CPB. Specimens were examined for apoptosis by flow cytometry analysis of annexin V-propidium iodide (PI) and Fas death receptor staining.

**Results:**

In the ModHT group, non apoptotic non necrotic cells (annexin negative, PI negative) decreased after CPB, while early apoptotic (annexin positive, PI negative) and late apoptotic or necrotic (PI positive) cells increased. In contrast, no change in the different cell populations was observed over time in the MiHT group. Fas expression rose after reperfusion in the ModHT group but not in MiHT patients, in which there was even a trend for a lower Fas staining after CPB (p = 0.08). In ModHT patients, a prolonged ischemic time tended to induce a higher increase of Fas (p = 0.061).

**Conclusion:**

Our data suggest that apoptosis signal cascade is activated at early stages during aortic valve replacement under ModHT CPB. This apoptosis induction can effectively be attenuated by a more normothermic procedure.

## 1. Background

Cardiomyocyte apoptosis has been involved in the pathophysiology of various cardiovascular diseases such as ischemic cardiomyopathy, hibernating myocardium, heart failure, reperfusion injury, and transplant rejection [1,2]. Recent reports have documented the prevalence of programmed cardiomyocyte death in open heart surgery under cardiopulmonary bypass (CPB), where it may contribute in parallel with necrosis to increase the bulk of myocardial death cells [3-12]. Apoptosis that occurs in this clinical setting can be induced by a wide variety of conditions and agents, including reactive oxygen-derived species, calcium and pressure overload, mechanical stress, nitric oxide, tumor necrosis factor, and angiotensin II [2,13]. It remains unclear whether apoptosis is a primary or a secondary event within cardiac surgery, and while some authors have related it to postoperative myocardial stunning [9] and non cardiac complications [3,4]; others have observed that inhibition of apoptosis has no impact on postischemic left ventricle functional recovery [14].

One of the reasons for scarce data concerning apoptosis and its pathophysiological consequences during cardiac surgery is the inferiority of the various techniques used to detect apoptotic cell changes. The most commonly used method, histochemical staining of the fragmented DNA by the TUNEL assay (*in situ *terminal deoxynucleotidyl-transferase mediated dUTP nick end-labeling), has a poor positive predicted value, as it labels not only fragmented DNA but also DNA in the process of repair as well as necrotic tissue [2,15]. Besides, the duration of the apoptotic program (12 to 24 hours) far exceeds the intraoperative window for sample acquisition (2 to 3 hours) [9], which render the observation of late signs of apoptotic cascade like DNA fragmentation useless. The investigation of early apoptotic signs, such as the translocation of membrane phospholipids or the activation of intracellular proteins, using more accurate tools may overcome these limitations.

Rationale for the use of hypothermia during cardiac surgery is based on its capacity to reversibly reduce metabolic activity in all cells and subcellular organelles, further limiting the rate of consumption of intracellular high-energy phosphates stores and ischemic injury [16]. Despite of this positive effect, hypothermia has also a deleterious influence on platelet function and increases citrate toxicity, with subsequent reduction in serum ionized calcium, leading to reversible coagulopathy, dysrhythmias, and depression of myocardial contractility [17,18]. The role of temperature for apoptosis is controversial and may be dual depending on the degree of hypothermia. While deep hypothermic circulatory arrest may activate apoptotic pathways [5], less aggressive hypothermia seems to inhibit them [19]. The temperature limits at which apoptosis is enhanced or attenuated should be accurately defined.

The purpose of this study was to investigate whether apoptosis occurs during elective aortic valve replacement for aortic stenosis and whether CPB temperature has any influence on cardiomyocyte apoptotic death rate. The reason for selecting non urgent isolated aortic valve patients was their greater homogeneity with respect to other cardiac pathologies, and the possibility of avoiding underlying causes of myocardial apoptosis other than cardiac surgery, such as ischemic cardiomyopathy or congestive heart failure. To adequate operative sample acquisition timing to the development of the apoptotic program we analyzed early stages apoptosis by flow cytometric analysis of annexin V and death receptor Fas staining.

## 2. Material and methods

### 2.1. Patients

After approval by our institution ethics committee on human research, informed consent was obtained from 20 patients who were scheduled for elective aortic valve replacement for aortic stenosis. Anesthesia was induced with fentanyl and etomidate, and was maintained with sevoflurane and fentanyl. Surgery was performed through a median sternotomy with standard CPB. Myocardial protection was obtained with intermittent retrograde administration of cold (4°C) blood cardioplegia (4:1 blood-to-crystalloid mixture). Patients were randomly assigned to one of two groups: moderate hypothermic CPB (ModHT group, n = 10) or mild hypothermic CPB (MiHT group, n = 10). After institution of CPB, patients in the ModHT group were cooled until core (nasopharyngeal) temperature reached 28°C, whereas the temperature of patients in the MiHT group was just let to drop until it leveled off spontaneously. Baseline biometric characteristics and CPB data of trial groups are shown in Table 1.

**Table 1:** Demographic, clinical, and CPB data of trial groups

	ModHT Group (n = 10)	MiHT Group (n = 10)	*p *Value
Age (y)	70.6 ± 9.7	67.7 ± 12.3	0.565
Female, n (%)	4 (40%)	4 (40%)	1.000
Hypertension, n (%)	6 (60%)	6 (60%)	1.000
Smoking history, n (%)	4 (40%	2 (20%)	0.626
Diabetes, n (%)	2 (20%)	1 (10%)	1.000
Hyperlipidemia, n (%)	4 (40%)	2 (20%)	0.626
Atrial fibrillation, n (%)	2 (20%)	4 (40%)	0.626
LVEF (%)	62.2 ± 17.1	57.8 ± 17.8	0.580
CPB time (min)	85.2 ± 11.3	87.5 ± 21.1	0.765
Aortic crossclamping time (min)	59.2 ± 7.8	59.0 ± 16.6	0.973
Minimal core CPB temperature (nasopharyngeal) (°C)	28.2 ± 1.1	34.5 ± 0.9	< 0.001
Need for postCPB inotropic support, n (%)	2 (20%)	2 (20%)	1.000

Results are expressed as means ± standard deviations, unless otherwise indicated.

CPB, cardiopulmonary bypass; LVEF, left ventricular ejection fraction.

### 2.2. Sample acquisition

Using a sharp scalpel, myocardial biopsy specimens (approximately 300 mm^3^) were obtained from the right atrial appendage of each patient before the start of CPB and after weaning from extracorporeal circulation. To minimize technical influences on apoptotic and necrotic measurements, particular care was taken not to mechanically irritate the site of atrial tissue harvesting with the forceps and with the venous cannula. Blood samples were also taken from the coronary sinus at the same time of heart biopsy collection for analysis of necrosis.

### 2.3. Isolation of cardiomyocytes

A single cell suspension was obtained from the heart tissue samples immediately after removal by mechanical disaggregation with a 50 μm filtered Medimachine system (BD Bioscience, CA, USA). Cells were washed with cold phosphate buffer saline (PBS) to exclude cell debris and resuspended in culture medium (RPMI 1640; Bio-Whittaker, Verviers, Belgium). This procedure rendered a preserved viability cell population as judged by trypan blue dye exclusion. To be sure that cardiac cells were specifically quantified, intracellular expression of the specific protein -connexin 43- was analyzed. After fixation and permeabilization with IntraStain kit (Dako Cytomation, Glostrup, Denmark), cells were incubated with connexin 43 rabbit polyclonal antibody (Zymed Laboratories, San Francisco, CA, USA). Binding was revealed by phycoerythrin conjugated goat anti-rabbit IgG (Jackson Immunoresearch, Baltimore, USA) and flow cytometry. The antibodies were initially titrated for optimal concentration, and cells incubation with only phycoerythrin-antibody was used as negative control.

### 2.4. Analysis of apoptosis

Apoptosis was measured by staining cells with a combination of fluoresceinated (FITC) annexin V and propidium iodide (PI) and by Fas death receptor quantification on cell membrane.

**Figure 1:**
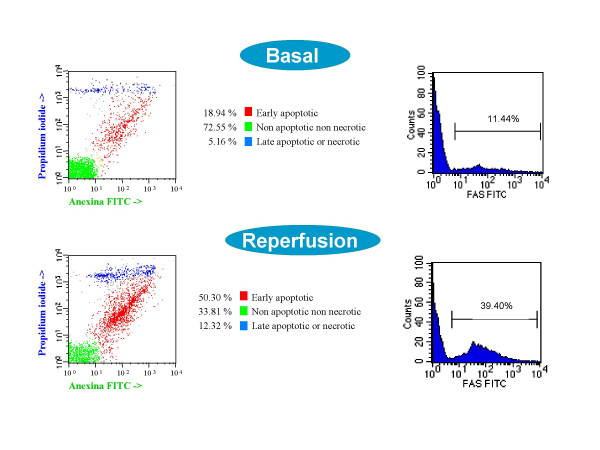
Flow cytometry analysis from a 75-year-old patient that underwent aortic valve replacement under ModHT CPB for aortic stenosis. From left to right (basal and reperfusion): dot-plot bidimensional diagram of annexin V-propidium iodide staining, and histogram showing Fas positive cardiomyocytes. Data are expressed as percentage of cells.

Changes in the plasma membrane are one of the earliest morphological features of apoptosis. In apoptotic cells, the membrane phospholipid phosphatidylserine is translocated from the inner to the outer leaflet of the plasma membrane, thereby exposing phosphatidylserine to the external cellular environment. Annexin V is a 35–36 kD Ca^2+^-dependent phospholipid-binding protein that has a high affinity for phosphatidylserine. Because externalization of phosphatidylserine occurs in the early stages of apoptosis, annexin V staining can identify apoptotic cells earlier than assays based on nuclear changes like DNA fragmentation, and precedes the loss of membrane integrity which accompanies the latest stages of cell death resulting from either apoptotic or necrotic processes. Therefore, staining with annexin V in conjunction with vital dye such as PI allows to identify early apoptotic cells (annexin positive, PI negative) and non apoptotic non necrotic cells (annexin negative, PI negative) (Figure 1). The assay does not identify between cells that have already completed the apoptotic program and those that have died as a result of a necrotic pathway, because in either case the dead cells will stain the same (annexin positive, PI positive). Briefly, the cells to analyze with the annexin V-PI method were resuspended in 100 μl of binding buffer (140 mM NaCl, 2.5 mM CaCl_2_, 10 mM HEPES; pH 7.4). Next, 5 μl of annexin V-FITC (Pharmingen, CA, USA) were added and incubated for 15 minutes at room temperature preserved of the light. Then, 100 μl of PI-PBS solution (50 mg/ml) (PI; Sigma, St Louis, MO, USA) were added and immediately data acquisition was made in the flow cytometer in order to avoid cell damage and the diffusion of PI through cell membrane.

Fas molecule (APO-1/CD95) belongs to the tumor necrosis factor receptor superfamily and may induce apoptosis by Fas-Fas ligand (Fas-L) binding. The Fas/Fas-L system is an essential part of the death receptor pathway regulating the induction of a rapid apoptosis independent of mitochondria. We measured Fas by membrane cell staining with FITC conjugated anti-CD95 monoclonal antibody (BD Biosciences) (Figure 1). Mouse IgG1 irrelevant antibody FITC conjugated was used as negative control of staining. After 15 minutes incubation at room temperature and dark, these cells were washed to remove the excess of antibody and fixed with 2% formaldehyde. Antibodies were used following manufacturer recommendations. In all cases, we have used a FACSort (BD Bioscience) flow cytometer to measure the cell-associated fluorescence with CellQuest and Paint-a-gate Pro analysis software (BD Bioscience).

### 2.5. Analysis of necrosis

Cardiomyocyte loss by necrosis was assessed by determination of creatine kinase, creatine kinase myocardial band, lactate dehydrogenase, troponin I, and lactate.

### 2.6. Statistical analysis

Data are presented as mean ± standard deviation. The χ^2^test and the two-tailed unpaired Student's *t *test were used when appropriate to compare the results between groups. Values from the different biochemical variables before and after CPB were compared using the two-tailed paired Student's *t *test. Correlation between the number of early apoptotic cells, Fas levels, and operative times was studied using Pearson's correlation coefficient. Differences were considered significant when *p *value was less than 0.05. Statistical analyses were performed with the software package SPSS for Windows, release 10.0.7 (SPSS Inc, Chicago, Ill, USA).

## 3. Results

Groups were well matched in terms of age, sex, prevalence of cardiovascular risk factors and atrial fibrillation, preoperative left ventricular function, and duration of CPB and aortic crossclamping (Table 1).

**Figure 2:**
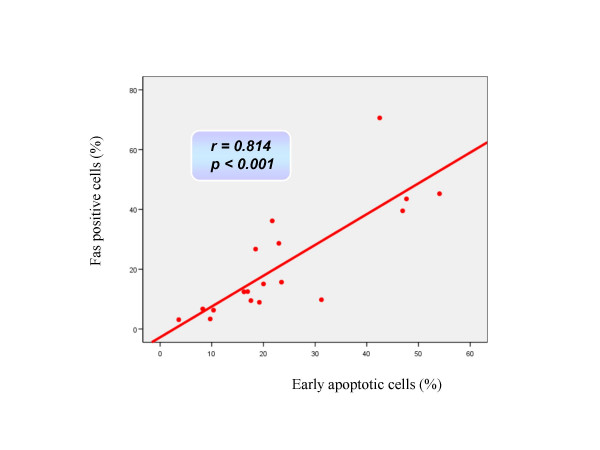
Correlation observed between the percentage of Fas positive cells and early apoptotic (annexin positive-PI negative) cells after CPB using Pearson's correlation coefficient.

**Figure 3:**
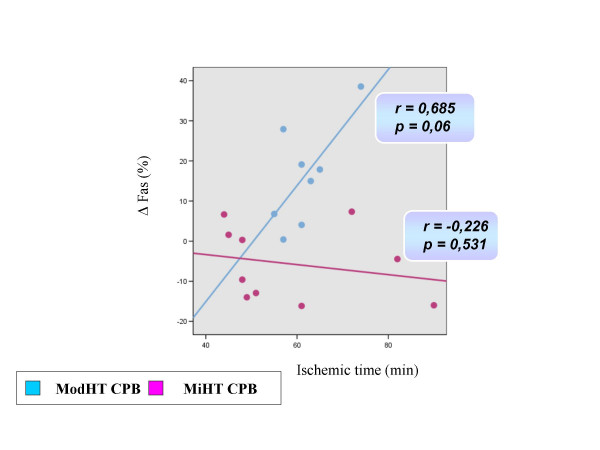
Correlation observed in both study groups between the rise of Fas over time (before CPB to reperfusion) and the duration of aortic crossclamping using Pearson's correlation coefficient.

Changes in cardiomyocyte death status during the operative procedure are summarized in Table 2. Apoptotic and necrotic parameters did not differ between groups before the start of CPB. In the ModHT group, non apoptotic non necrotic cells (annexin negative, PI negative) decreased immediately after CPB, while early apoptotic (annexin positive, PI negative) and late apoptotic or necrotic (PI positive) cells increased. In contrast, no change among the different cell populations for apoptosis or necrosis was observed over time in the MiHT group. ModHT patients exhibited a higher percentage of early apoptotic cells and a lower number of live cardiomyocytes than MiHT patients after CPB. Fas expression rose after reperfusion in the ModHT group but not in the MiHT, in which there was even a trend for a lower Fas staining at the end of CPB (p = 0.08). A significant correlation was observed between the Fas level and the percentage of annexin positive-PI negative cells after CPB (r = 0.814, p < 0.001) (Figure 2). In the ModHT group, a prolonged aortic crossclamping time tended to induce a higher increase of Fas over time (r = 0.685, p = 0.061) (Figure 3). Molecular markers of myocardial necrosis increased after CPB in a similar way in both groups, with the exception of lactate dehydrogenase that did not change consistently after reperfusion, and creatine kinase myocardial band that rose only in the MiHT group.

**Table 2:** Cell death status over time

	Basal (ModHT group/MiHT group)	Reperfusion (ModHT group/MiHT group)	*p *Value (ModHT group/MiHT group)
Non apoptotic non necrotic cells *(annexin negative, PI negative) *(%)	73.86 ± 18.49/67.28 ± 16.40	51.20 ± 23.88/71.71 ± 11.35 ^a^	0.002/0.307
Early apoptotic cells *(annexin positive, PI negative) *(%)	16.45 ± 13.36/20.70 ± 12.21	33.21 ± 17.85/17.62 ± 8.06 ^a^	0.003/0.180
Late apoptotic or necrotic cells *(annexin positive, PI positive) *(%)	6.97 ± 3.68/10.49 ± 5.92	11.45 ± 5.11/8.90 ± 5.36	0.009/0.513
Fas positive cells (%)	15.14 ± 17.85/20.03 ± 14.99	31.34 ± 22.54/14.30 ± 10.66	0.009/0.081
CK (IU/L)	53.1 ± 21.6/60.2 ± 23.7	167.1 ± 158.5/153.8 ± 87.9	0.045/0.011
CK-MB (IU/L)	29.13 ± 18.20/20.00 ± 3.62	42.25 ± 33.80/37.70 ± 12.18	0.355/0.001
LDH (IU/L)	363.0 ± 94.2/334.5 ± 61.6	385.4 ± 152.7/363.5 ± 80.1	0.723/0.240
Troponin I (μg/L)	0.32 ± 0.33/0.22 ± 0.15	1.48 ± 1.37/1.45 ± 0.53	0.029/< 0.001
Lactate (mmol/L)	1.53 ± 0.88/1.62 ± 0.91	3.60 ± 1.32/3.19 ± 1.19	0.002/0.005

Results are expressed as means ± standard deviations.

^a ^p < 0.05 compared to ModHT group.

CK, creatine kinase; CK-MB, creatine kinase myocardial band; LDH, lactate dehydrogenase; PI, propidium iodide.

## 4. Discussion

Apoptosis has been reported not only to occur during open heart surgery but also to be related to postoperative myocardial stunning, renal insufficiency, and cerebral dysfunction [3,4,9]. Cardiac surgery combines two pro-apoptotic stimuli: ischemia-reperfusion injury and CPB. Both processes lead to the activation of vascular endothelial cells and neutrophils, thereby stimulating cellular calcium overload, and the generation of reactive oxygen-derived species and cytokines that may further initiate the apoptotic program in cardiomyocytes [2,13]. Serum of patients after CPB has been shown to have a strong proapoptotic activity on human endothelial cells [7]. Several recent studies have demonstrated that both the mitochondrial and the death receptor pathways of caspase activation are triggered during open heart surgery leading to myocardial apoptosis. Schmitt and coworkers found a postreperfusion highly significant 3.4 ± 0.4-fold increase of the mitochondrial respiratory chain protein -cytochrome c- in the cytosol of atrial myocytes during coronary artery bypass grafting [9]. Activation of caspases-3 and -7 and an elevated release of soluble Fas and Fas ligand have also been detected at the end of extracorporeal circulation [6,8,10].

Strategies towards a less apoptogenic procedure appear to be a goal to achieve excellence in cardiac surgery and may include shortening of ischemic and CPB times, increased use of intraaortic balloon pump and noncatecholamine inotropes, addition of antiapoptotic medication, or modulation of intraoperative temperature.

Hypothermia improves resistance to ischemia-reperfusion injury in the myocardium. Protection afforded by low temperature may be linked to a wide variety of effects, including reduction of ATP store depletion, inhibition of endothelial cell expression of E-selectin, modification of cell death signaling pathways, and preservation of gene expression for mitochondrial proteins [16]. Nevertheless, the impact of intraoperative hypothermia on cardiomyocyte apoptosis during cardiac surgical procedures remains controversial. Cooper and colleagues have observed an increased rate of apoptosis in duodenal and myocardial tissue from pigs subjected to deep hypothermic circulatory arrest as compared to normothermic CPB controls [5]. Other authors, on the contrary, have assessed by gene array analysis a protective effect of hypothermia (30°C) on the cardioplegia-arrested rabbit heart, based on its ability for decreasing the expression of proapoptotic proteins (p53, *bak*) and promoting antiapoptotic factors (*Bcl-x*) [19]. This finding is also supported by studies including experimental models of cerebral hypoxia in which hypothermia mitigated post-ischemic programmed cell death [20-22].

Our results suggest an additive pro-apoptotic effect of moderate hypothermia that may superimpose on the *per se *apoptogenic state of CPB, and that might be involved in the decreased systolic performance often observed with cooling [17,18]. Two early reversible morphological features of apoptosis signal cascade activation, phosphatidylserine externalization and Fas expression in the plasma membrane, increased after reperfusion in the ModHT group but not in MiHT patients.

The annexin V staining as a method for cell death analysis has been mainly applied in lymphoid disorders, but there is increasing evidence that it is also useful for the detection of apoptotic cardiomyocytes [1,23,24]. When compared to the TUNEL assay, both methods have shown a similar sensitivity and specificity [15]. Scholz and Leaes have recently employed annexin V technique for the analysis of neutrophil and lymphoid apoptosis that occurred during CPB in pigs and humans, respectively [11,12]. Annexin V staining reveal phosphatidylserine outer leaflet membrane exposure, which is one of the earliest hallmarks of cells undergoing apoptosis [1,15,23,24]. Annexin positive cardiomyocytes retain contractile function but exhibit a decreased cell width indicative of cell shrinkage, and a markedly elevated mitochondrial free Ca^2+ ^[24]. In our study, annexin V positive-PI negative cells were detected in both groups before the start of CPB, but whereas in the ModHT group the number of cardiomyocytes that had initiated the apoptotic program increased after reperfusion, in MiHT patients it remained unchanged.

The biological relevance of Fas has been mainly proved in relation to T lymphocytes activity in the context of autoimmune and hematopoietic diseases [6,8]. Nevertheless, Fas is also expressed in other non lymphoid cells such as cardiomyocytes or hepatocytes [25]. In fact, it has been shown to be involved in the pathophysiology of congestive heart failure, graft rejection, end-stage cardiomyopathy, and myocarditis [6]. Kawahito and Joashi have observed elevated serum levels of the soluble form of Fas and Fas ligand after completion of cardiac surgical procedures under moderate hypothermic CPB [6,8]. They also found prolonged operative times to induce higher serum concentrations of Fas. In the present study, a significant but similar percentage of cardiomyocytes were Fas positive before CPB in both groups. A higher Fas expression was observed after reperfusion in ModHT patients, but not in those operated under mild hypothermia, which tended to show less Fas positive cells after weaning from CPB. In addition, in the ModHT group there was a trend for a higher increase in Fas staining with longer ischemic times. Phanithi and colleagues have also demonstrated that mild hypothermia (33°C) reduced Fas expression in a rat model of reversible cerebral ischemia when compared to normothermic controls [22]. We measured the membrane-bound form of Fas, which is known to be of far greater importance in the apoptotic process than the soluble form of the molecule that was determined in previous studies [6,8].

In conclusion, our data show that apoptosis signal cascade is activated at early stages in human myocardium during aortic valve replacement under ModHT CPB. This apoptosis induction can effectively be attenuated by a near-normothermic strategy. In our experience, the biochemical benefit of mild hypothermia is not apparently based on the reduction of bypass times or the use of less postoperative inotropic support, and should response to a direct impact of temperature on cell membrane stability and death receptor signaling pathway.

### 4.1. Limitations of the study

Heart tissue samples were harvested from the right atrial appendage with the intention of obtaining an appropriate amount of tissue without impairing cardiac function. This strategy has been previously reported for the study of apoptosis during CPB [9]. Nevertheless, the distribution of the cardioplegia infused through the coronary sinus is not uniform in the entire heart, and left cavities may be better preserved than the right ones. The hypothesis that our findings in the right atrium mirror changes in the left ventricular muscle must be made with cautious and represents a limitation of the present work.

The percentage of cardiomyocytes that have initiated the apoptotic program at the time of first sample acquisition (preCPB) seems remarkably high in both groups (more than 16% in annexin assay, and more than 15% in Fas staining), which may indicate a lack of specificity of both techniques. Although tissue damage during biopsy collection might have contributed to artifact the results, we hypothesize that our data may reliably reflect the state of apoptosis in atrial myocardium just before CPB. In fact, contributing factors such us anesthesia induction, sternotomy, or cannulation may trigger the apoptotic cascade before the start of CPB, as it has been suggested by previous works [10]. Besides, apoptosis is a reversible process until the downstream caspases (caspase-3, -6, -7) are activated [2], so not all the annexin positive-PI negative and Fas positive cells will necessarily complete the apoptotic program and die.
